# Growth and Neurodevelopmental Outcomes of Children With Congenital Hypothyroidism Identified Through Dried Blood Spot Newborn Screening at a Tertiary Care Hospital in Pakistan

**DOI:** 10.1002/edm2.70296

**Published:** 2026-08-22

**Authors:** Bushra Rehman, Muzna Arif, Abida Akbar, Nadia Mazhar, Zahra Hoodbhoy, Fozia Memon, Muhammad Nasheet Sagri, Sumaiyah Zahid, Hafsa Majid, Farzana Faqiruddin, Salman Kirmani, Khadija Nuzhat Humayun

**Affiliations:** ^1^ Aga Khan University Hospital Karachi Pakistan; ^2^ Department of Computer Sciences National University of Computer and Emerging Sciences, FAST NUCES Karachi Pakistan

**Keywords:** congenital hypothyroidism, dried blood spot, growth, neurodevelopmental outcomes, newborn screening, Pakistan

## Abstract

**Background:**

Congenital hypothyroidism (CH) is the most common congenital endocrine disorder and a leading preventable cause of intellectual disability in children. Pakistan lacks a national NBS program, and CH incidence is estimated at 1:1000–1:600, higher than the global average. AKUH, Karachi, introduced DBS screening for CH in 2019. This study assessed outcomes in neonates screened between 1 April 2019 and 31 March 2024.

**Methods:**

Neonates with positive DBS (TSH > 10 μIU/mL) and confirmed CH (FT4 < normal and TSH > 20 μIU/mL) were included; growth and ASQ‐3 records were extracted.

**Results:**

Among 53,799 screened babies, 971 were screened positive; of these, 40 (4.12%) were true positives. Thirty‐nine neonates were included; 34 had ASQ‐3 records. Females were *n* = 23 (59%); median gestational age was 38 weeks, 82.1% had normal birth weight, and median birth weight was 2.8 kg. DBS was performed after 36 h in 64.1%. Eight neonates were symptomatic; prolonged jaundice occurred in 20.51%; one had trisomy 21; and eight (20.5%) had a family history of hypothyroidism. At 3 years, weight SDS ranged from −2.02 to 0.98 and height SDS from −2.15 to 1.65, with median height SDS of −0.62. Global delay was observed in one child (3%), fine motor and personal‐social impairment in one child each (3%) and normal development in 32 (94.1%).

**Conclusions:**

The child with global delay was suspected to have an underlying neurometabolic disorder unrelated to CH. DBS screening at AKUH detected CH consistently with local estimates, and early treatment was associated with normal growth and neurodevelopment in most neonates at 2‐year follow‐up. These findings support NBS expansion in Pakistan.

## Graphical abstract

Hospital‐based dried blood spot newborn screening enabled early diagnosis and timely levothyroxine treatment of congenital hypothyroidism, resulting in preserved growth and normal neurodevelopment in most children. These findings support expansion of newborn screening programs in Pakistan.

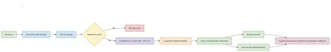

## 1 Introduction

Congenital hypothyroidism (CH) is the most common congenital endocrine disorder and the most common preventable cause of intellectual disability in children [1]. Thyroid hormone is essential for normal brain development; deficiency during critical early developmental windows results in brain damage and cognitive impairment [2]. Unrecognized CH is described as the most common cause of preventable cognitive delay, yet affected infants often appear normal and asymptomatic at birth, making clinical detection unreliable without systematic screening [3]. Newborn screening (NBS) has largely eliminated the severe physical and neurodevelopmental consequences of untreated CH in countries where it is routinely implemented [4]. Despite the proven value of NBS, enormous global gaps persist. A recent comprehensive assessment found that only 29.6% of children worldwide are screened for CH, and approximately 70% of newborns lack access to NBS, predominantly in African and Asian countries [5]. Pakistan, the world's fifth most populous country with 241.5 million people and 36.57% of the population under 14 years of age, lacks a national NBS program. The establishment of NBS in developing countries poses major challenges, as it competes with other health priorities, including control of infectious diseases, malnutrition and immunization programs [6]. The neurodevelopmental prognosis of CH is critically dependent on early detection and treatment. Early and adequate levothyroxine treatment results in excellent neurodevelopmental outcomes for most patients with CH [1], while delayed diagnosis and treatment are consistently associated with worse cognitive and developmental outcomes across diverse settings [7, 8]. Higher initial levothyroxine doses and rapid normalization of thyroid function have been linked to better full‐scale IQ scores, [9] and ASQ‐based developmental screening in early‐treated CH cohorts has shown outcomes comparable to healthy controls when treatment is initiated within the first month of life [10]. Despite these advances, evidence from Pakistan on long‐term growth and neurodevelopmental outcomes of NBS‐detected CH children remains scarce. The objective of this study was to determine the growth and neurodevelopmental outcomes of children with CH identified through DBS‐based NBS at AKUH over 5 years (1 April 2019 to 31 March 2024).

## 2 Methods

### 2.1 Study Design and Setting

This was a single‐centre retrospective observational cohort study, conducted at Aga Khan University Hospital (AKUH), Karachi, Pakistan, a tertiary care centre serving both urban and rural patients. Most females registered for delivery come from urban areas, while complicated cases from rural and peripheral regions are also referred. Neonates are routinely discharged after 48 h; DBS samples are obtained right before discharge as part of the institutional NBS protocol introduced in 2019 [11]. Patient confidentiality was maintained using codes and serial numbers instead of patient identifiers; all data were stored in password‐secured Forms. Neonatal screening for CH was initiated at Aga Khan University Hospital (AKUH) in Karachi in March 1987; by April 1988, 5000 neonates had been screened with five confirmed CH cases and an estimated incidence of 1 per 1000 newborns [12]. More recent data from the AKUH dried blood spot (DBS)‐based screening program (2019–2022) show high operational coverage: 30,402 neonates were screened with total NBS coverage of 96.2% and an observed CH incidence of 1:1126. The positive screen cut‐off for DBS‐TSH was set at > 10 mIU/L, and CH was confirmed when serum TSH was ≥ 20 uIU/mL along with low free thyroxine (FT4), at which point levothyroxine replacement therapy was initiated [11]. Patient confidentiality was maintained using codes and serial numbers instead of patient identifiers; all data were stored in password‐secured Forms.

### 2.2 Eligibility Criteria

All neonates who screened positive on DBS and were subsequently confirmed to have CH by serum testing between 1st April 2019 and 31st March 2024 were included. Exclusion criteria uncluded all neonates with dysmorphism, and developmental delays due to other causes, preterm babies less than 34 weeks of gestation.

### 2.3 Operational Definitions

Newborns were classified as screen‐positive if their dried blood spot (DBS) TSH exceeded 10 μIU/mL. Screen‐positive neonates underwent confirmatory serum testing, and congenital hypothyroidism (CH) was confirmed when free thyroxine (FT4) fell below the institutional normal range (0.86–2.49 ng/dL) with a concurrent serum TSH greater than 20 μIU/mL, at which point levothyroxine replacement therapy was initiated. Confirmed cases were subsequently categorized as either transient or permanent CH based on disease course: transient CH was defined as hypothyroidism that resolved following a supervised off‐treatment trial, typically undertaken at approximately 3 years of age, allowing successful discontinuation of levothyroxine; permanent CH was defined as hypothyroidism confirmed to require lifelong levothyroxine replacement.

### 2.4 Data Collection

All confirmed CH cases were identified from the institutional screening database. A structured proforma was completed from laboratory results and patient files. Growth parameters (weight and height) were extracted from routine follow‐up records. Neurodevelopmental assessment scores records were extracted from files, where the ASQ assessment is routinely performed in clinic visits. Neurodevelopmental status was assessed in clinics using the Ages and Stages Questionnaire, Third Edition (ASQ‐3), a validated parent‐completed screening tool for children from birth to 60 months [10, 13]. The ASQ‐3 comprises 30 items across five developmental domains: communication, gross motor, fine motor, problem‐solving and personal‐social skills. Each item is scored as Yes (10 points), Sometimes (5 points), or Not yet (0 points). Domain scores are plotted on a reference chart with white (above cutoff/normal), grey (close to cutoff/monitoring zone) and black (below cutoff/referral zone) areas [13, 14]. The ASQ has been validated against the Battelle Developmental Inventory, 2nd Edition (BDI‐2) and the Bayley Scales of Infant Development, 2nd Edition (BSID‐II), demonstrating 100% sensitivity and 87% specificity at 24 months [14]. Due to the small sample size and variability in patient ages at follow‐up, the last recorded ASQ‐3 assessment for each child was used for analysis. Neurodevelopmental outcome was defined as normal if all five domain scores lay within the reference range. Impairment in a single domain was classified as communication impairment, gross motor function impairment, fine motor function impairment, problem‐solving skills impairment, or personal‐social skills impairment, depending on the affected domain. Global delay was defined when more than two domain scores fell below the reference range.

### 2.5 Statistical Analysis

Descriptive statistics were used to summarize screening data, demographic characteristics, clinical features, laboratory parameters and neurodevelopmental outcomes. Continuous variables were presented as medians along with min‐max, while categorical variables were expressed as frequencies and percentages. All statistical analyses were performed using IBM SPSS Statistics version 23.0 (IBM Corp., Armonk, NY, USA).

## 3 Results

**FIGURE 1:**
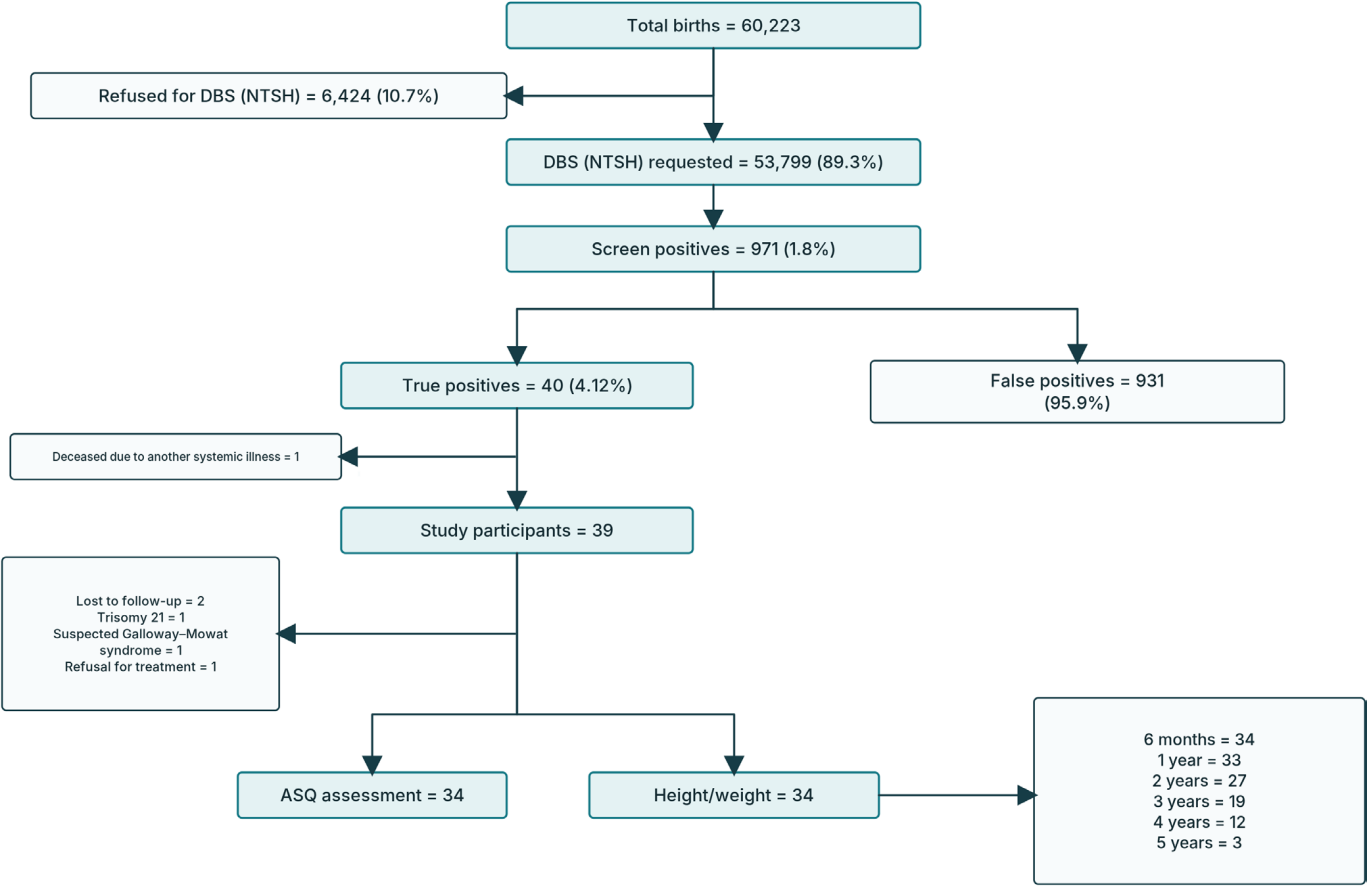
Flowchart showing total DBS positive rate.

**FIGURE 2:**
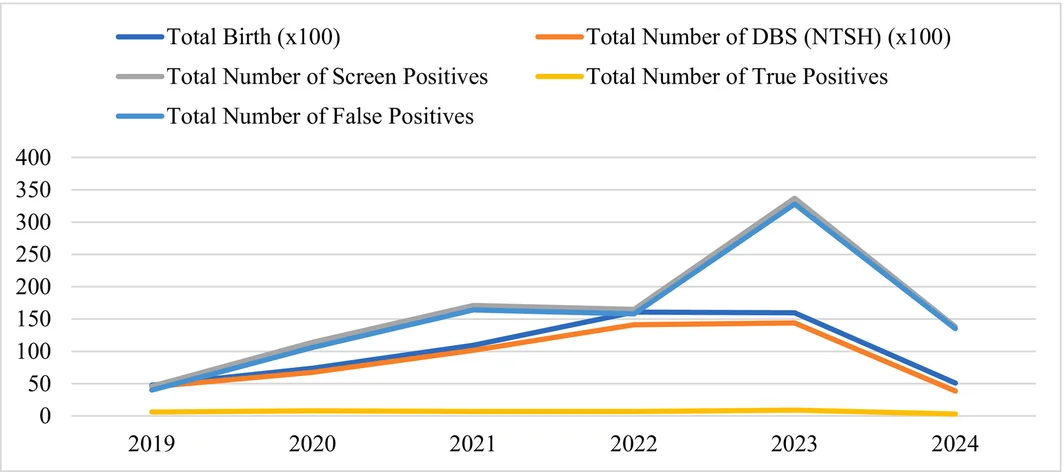
Trends in DBS‐NTSH newborn screening coverage and outcomes, 2019–2024.

During the study period, 60,223 babies were born; among them, 53,799 (89.33%) received DBS‐NTSH screening, of whom 971 (1.8%) were screen positive. Nearly 10.7% (*n* = 6424) refused DBS, with the commonest reason being domestic or financial. Of the 971 screen positive neonates, 40 (4.12%) were true positives, yielding an observed CH incidence of approximately 1:1345 (40/53,799). Out of 40 true positive neonates, one neonate died due to another systemic illness; hence our study includes 39 neonates. Figure 1 provides a visual representation of the patient selection and exclusion process, leading to the final group of 39 patients, whose clinical characteristics were analysed in the study. ASQ‐3 data were available for 34 of 39 participants; five were excluded due to loss to follow‐up, Trisomy 21, treatment refusal and suspected Galloway–Mowat syndrome. Growth data were similarly available for 34 participants, with numbers declining from 33 at 6 months to 3 at 5 years. From 2019 to 2023, the percentage of true positives was highest in 2019 (13.0%), followed by 7.0% in 2020 (Table 1). The rate of screen positive rates increased over time; however, the proportion of true positives decreased, with false positives accounting for over 90% of positive screens in most. There was a noticeable increase in screen positive cases in 2024, as shown in Figure 2.

**TABLE 1:** Baseline characteristics of neonates with congenital hypothyroidism.

Variable	*N* (%)/Median (Min–Max)
Gender	
Female	23 (59%)
Male	16 (41%)
DBS screen hour	
24–36	14 (35.89%)
> 36	25 (64.1%)
Gestational Age (week)	38 (34–41.29)
Gestational Age categories	
34–36	10 (25.64%)
37–42	29 (74.35%)
Birth weight (kg)	2.8 (1.8–3.7)
Birth weight categories	
< 2.5	7 (17.9%)
2.5–4	32 (82.1%)
Maternal hypothyroidism^a^	8 (20.5%)

^a^ All mothers were on thyroxine.

### 3.1 Characteristics of CH Population

The baseline characteristics of these neonates are given in Table 1. Of 39 participants, the majority were female (*n* = 23, 59%). The average gestational age was 37.9 ± 1.7 weeks with mostly neonates born between 37 and 42 weeks, and only 10 (25.64%) neonates were preterm (34–37 weeks), but none of the neonates in our cohort was born at < 34 weeks of gestation. The average birth weight was 2.77 ± 0.45 kg, and mostly had normal weight (*n* = 32, 82.1%). Nearly 20.5% (*N* = 8) mothers had hypothyroidism, and the same proportion reported using thyroxine. No history of hyperthyroidism or the use of anti‐thyroid drugs was reported in mothers. Mostly neonates had DBS screen hours greater than 36 (*N* = 25, 64.1%), while the rest were performed between 24 and 36 h of life. Eight (20.51%) neonates had a family history of hypothyroidism, including six first‐degree relatives and two second‐degree relatives, while only two neonates (5.12%) had a family history of congenital hearing loss.

### 3.2 Laboratory and Clinical Profile

**FIGURE 3:**
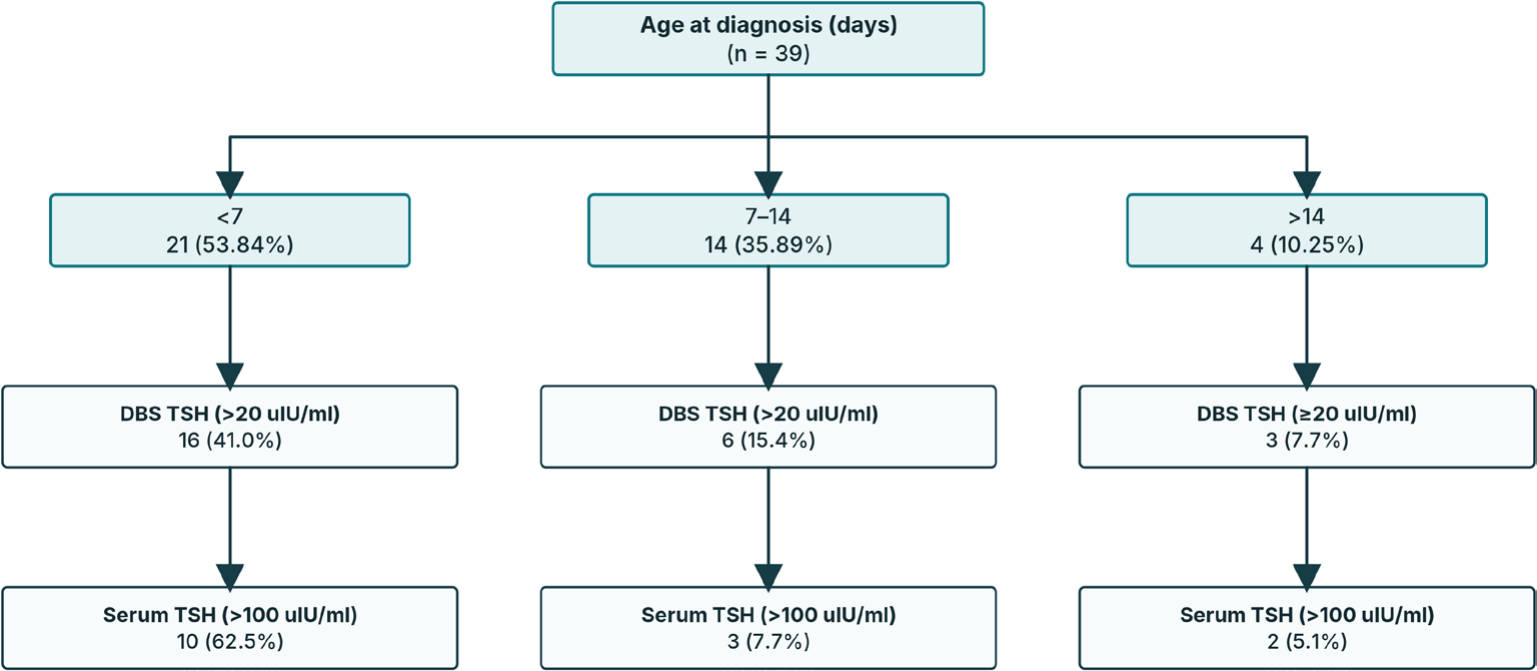
Flowchart showing the categorization based on age at diagnosis.

Most neonates were diagnosed within 14 days of life (*n* = 35, 89.73%), with the majority diagnosed before 1 week of life (53.84%). Four neonates (10.25%) were diagnosed after the 14th day of life. One neonate initially had a mildly elevated DBS‐TSH of 14.6 μIU/mL with a serum TSH of 13.5 μIU/mL and normal FT4; however, serial monitoring revealed a progressive rise in TSH to 19.6 μIU/mL and subsequently 23 μIU/mL, accompanied by declining FT4, prompting initiation of levothyroxine therapy. Two other neonates were diagnosed after 14 days due to initial parental refusal to repeat serum TSH testing. The fourth neonate's parents refused both confirmatory testing and treatment initiation; despite maximal counselling, they were lost to follow‐up and returned for repeat TSH testing at the 300th day of life, by which time the child was already neuro‐developmentally delayed. Neonates diagnosed earlier (< 7 days) tend to have a higher range of DBS TSH > 16 uIU/mL (*n* = 16, 41.02%) and serum TSH > 100 uIU/mL (*n* = 10, 62.5%) while, among those who were diagnosed later (7–14 days), only 6 (15.4%) had DBS TSH (> 20 uIU/mL) and 3 had serum TSH (> 100 uIU/mL) and FT4 (< 0.86 ng/dL) shown in Figure 3.

The clinical and laboratory features at diagnosis are summarized in Table 2. Most neonates (*n* = 30, 76.9%) were asymptomatic; among the nine symptomatic neonates (23.1%), prolonged jaundice was the commonest presentation (*n* = 8, 20.51%). Treatment was initiated within 14 days of life in the majority (*n* = 34, 87.17%), with delayed initiation in the remainder attributable to delayed TSH surge, late confirmatory testing, or parental hesitancy. At 3 years, four children were successfully weaned off thyroxine, while two failed the off‐treatment trial and required lifelong replacement. Overall compliance was high (*n* = 35, 89.74%). Overall, 28 neonates (71.79%) maintained regular follow‐up as per European Society of Paediatric Endocrinology guidelines, which recommend follow‐up 1–2 weeks after initiation of levothyroxine, then every 2 weeks until TSH normalizes; thereafter every 1–2 months until 6 months of age, every 2–3 months from 6 to 12 months, every 3–4 months from 1 to 3 years and every 6–12 months thereafter.

**TABLE 2:** Clinical and laboratory features of babies at diagnosis.

Variables	*N* (%)
Thyroxine started on which day of life (DOL)	
< 7	9 (23.07%)
7–14	25 (64.1%)
> 14	5 (12.82%)
Starting dose of thyroxine in mcg/kg	
< 10	7 (17.94%)
10–15	32 (82.05%)
Dose at Age 1 Year (mcg/kg)	
> 2.4	30 (76.92%)
≤ 2.4	9 (23.07%)
Off‐treatment trial at 3 years	
Failed	2 (5.29%)
Successful	4 (10.25%)
Not applicable^a^	33 (84.61%)
Compliance with thyroxine	35 (89.74%)
Follow‐up Status	
Regular	28 (71.79%)
Irregular	11 (28.20%)

^a^ On higher dosage/dose increased over time.

### 3.3 Growth Outcomes

Furthermore, as shown in Figure 1, most children were assessed at 6 months, but we included growth parameter at 3 years of age to see the long term outcomes. At 3 years, the average weight SD of these participants was −0.57 (±0.89) and average SD height was −0.4 (±1.03). The average overall weight SDS was −0.98 (±1.05) at 6 months, where female neonates (−1.02 ± 1.11) reported comparatively lower weight score as compared to male (−0.93 ± 0.99). The weight and height SD scores are shown in Table 3.

**TABLE 3:** Weight and height standard deviation scores for children with congenital hypothyroidism.

		6 months	1 year	2 years	3 years	4 years	5 years
Weight SD	Overall	−1.05 (−2.71, 1.58)	−0.52 (−2.94, 2.4)	−0.55 (−3.38, 0.71)	−0.58 (−2.02, 0.98)	−0.8 (−1.87, 1.02)	−1.46 (−2.01, −1.42)
Height SD	Overall	−0.97 (−3.13, 1.58)	0.09 (−2.19, 2.11)	−0.1 (−2.66, 2.47)	−0.62 (−2.15, 1.65)	−0.7 (−1.98, 0.31)	−1.46 (−1.67, −0.98)
Overall sample size for each time point	*n*	34	33	27	19	12	3

Children with congenital hypothyroidism maintained weight and height SD scores close to the reference mean throughout the first 5 years, with height and weight SD scores reflecting largely preserved linear growth with early treatment.

### 3.4 Neurodevelopmental Outcomes

**FIGURE 4:**
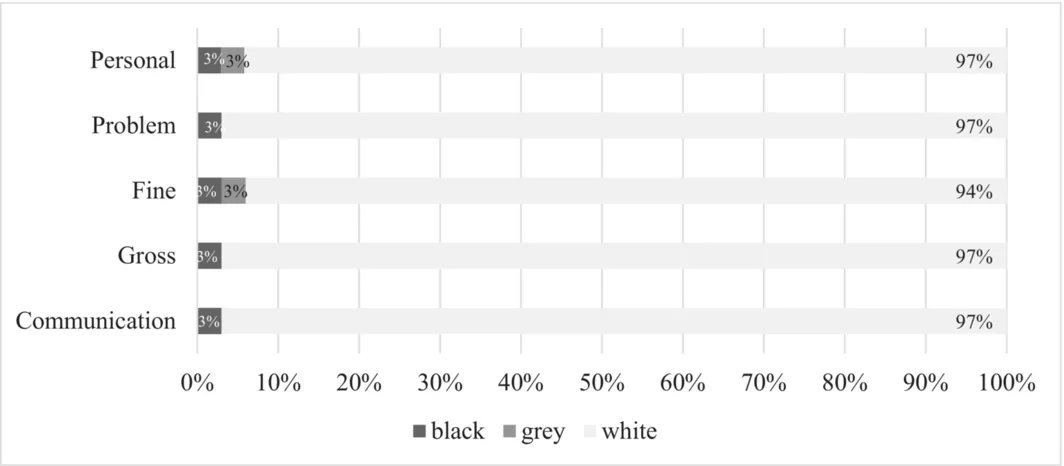
Neurodevelopmental outcomes across each domain measure using ASQ‐3 (Black: Global Delay, Grey: Impairment in at least one domain, White: Normal Development).

Outcomes of the ASQ‐3 are visualized in Figure 4. The majority of the neonates demonstrated normal neurodevelopment across all domains. Global delay was observed in 1 child (3%), and fine motor and personal‐social impairment in one child (3%), while normal development was observed in 94.1%. (*n* = 32). The child with global delay was born at 36 weeks of gestation with low birth weight, without any history of perinatal asphyxia, he was referred to neurology because of developmental delay despite adequate control and compliance; and he was suspected to have an underlying neurometabolic disorder unrelated to CH. The laboratory profile of these two neonates showed that both had severe congenital hypothyroidism, as evidenced by serum TSH of above 60 uIU/ml in both, with markedly low FT4.

## 4 Discussion

This study reports the growth and neurodevelopmental outcomes of children with CH identified through DBS‐based NBS at a tertiary care hospital in Pakistan over 5 years. These data represent one of the few outcome assessments from a structured CH screening program in a Pakistani setting, where national NBS coverage remains absent and hospital‐based programmes serve as the primary vehicle for early CH detection.

Pakistan lacks a national NBS program, and implementation competes with other health priorities and practical constraints [6]. Against this backdrop, AKUH's experience demonstrates a notably high CH incidence. Since launching in 1987 with 5000 neonates screened in its first year, [12] the program has evolved to DBS‐based screening (2019–2022), achieving 96.2% coverage with a DBS‐TSH cut‐off > 10 μIU/mL and confirmatory criteria of serum TSH ≥ 20 μIU/mL with low FT4 [11]. CH incidence across both periods—1:1000 initially and 1:1126 more recently—is notably higher than the global estimate of 1:2000–1:3000, consistent with elevated consanguinity rates and iodine deficiency in the region [5, 6, 11, 12].

In a national survey, 90.6% of Pakistani clinicians believed CH screening was essential, yet only 41.3% offered it in practice; barriers included cost of testing (32.2%), non‐availability of testing services (8.5%) and lack of implemented screening policies or national guidelines (5.8%) [15]. Demand‐side barriers also exist: among pregnant women in Karachi, baseline awareness of CH and its implications was low, but improved substantially (from 20% to approximately 98%) after a health education intervention, alongside increased willingness to opt for newborn screening (57.7% pre‐intervention to 78.9% post‐intervention; relative risk 1.38, *p* < 0.0001) [16]. Notably, even within the AKUH programme, the recall rate for confirmatory testing was only 63.13%, [11] underscoring that the principal bottleneck may lie not in screening coverage but in confirmatory testing completion and longitudinal follow‐up adherence.

In our cohort, children with CH maintained weight and height SD scores close to the reference mean throughout the first 5 years of life. Height SD scores remained near zero from 6 months to 3 years reflecting largely preserved linear growth, while weight SD scores similarly stayed within an acceptable range. A mild decline in both parameters was observed at age 5, although this observation is based on only three children and should be interpreted with caution. The mild decline in both parameters at age 5 warrants continued monitoring, but overall these findings demonstrate favourable growth outcomes in children with congenital hypothyroidism managed with early and adequate thyroid hormone replacement. Female neonates had slightly lower weight SD scores than males at 6 months, though the small sample size precludes meaningful sex‐based comparisons. Overall, these findings indicate that timely diagnosis and levothyroxine replacement supported near‐normal growth trajectories during this critical developmental period.

Our findings can be contextualized with the international literature on growth in treated CH. In a large Iranian longitudinal cohort of 760 neonates, growth in height and weight was significantly correlated with age at onset of treatment, initial levothyroxine dose and age at thyroxine normalization, suggesting that earlier treatment and appropriate dosing optimize growth trajectories [17]. In a Qatar‐based cohort of 45 children diagnosed through national screening and followed for at least 6 years, statural growth was normal compared with reference data, and the authors concluded that effective screening and treatment completely assures normal linear growth in CH [18]. Similarly, a randomized comparison of two initial levothyroxine dosing schemes (10–12.5 vs. 12.6–15 μg/kg/day) found that weight and height were normal and comparable between groups throughout follow‐up [19].

However, population‐based data suggest that residual growth differences may persist. In a Korean national birth cohort (*n* = 919,707), short stature at 66–71 months (height‐z < −1.63) was increased in children with CH compared with peers (adjusted RR 2.27, 95% CI: 1.328–3.865), while obesity was not increased [20]. A complementary nationwide longitudinal analysis of 408 CH patients similarly reported reduced height‐for‐age z‐scores and increased risk of low height‐for‐age z‐score, without elevated BMI‐for‐age z‐score [21]. An Indian study further demonstrated that initiation of levothyroxine within the first 3 months of life is critical for preserving linear growth, cranial development and skeletal proportionality [22]. These findings collectively indicate that normal growth is achievable in most early‐treated CH children. Our cohort included children of variable ages, and the long‐term outcomes of growth should be monitored for better interpretation and correlation with the literature reported globally.

Our ASQ‐3 findings can be compared to several international cohorts that used the same instrument. Two Iranian cohorts using ASQ reported substantially higher rates of developmental delay than our findings: Razavi et al. [13] found 41% delay in 78 children, with gross motor as the most affected domain, and Mirzarahimi et al. [23] reported a similar 41% impairment rate in Ardabil; in both studies, outcomes correlated with age at diagnosis and treatment initiation. In our cohort, we observed 6% developmental delays compared to the data reported, but our cohort included only 39 children from a single centre and a larger sample size is needed for better comparison.

Conversely, when treatment is initiated early and at adequate doses, ASQ outcomes can be reassuring. Nafei et al. compared 40 early‐treated CH neonates (treated within the first month with levothyroxine 10–15 μg/kg/day) against 40 healthy controls and found no significant difference in mean ASQ scores, concluding that developmental skills were at standard levels. This goes very well with our cohort, as we have reassuring ASQ‐3 scores for the majority of children, who were started on treatment before 14 days of life [10]. Nazari et al., using a combined assessment approach (ASQ + Bayley + WPPSI) in 34 CH children versus 68 controls, similarly found no significant difference in physical or mental growth [24].

Large‐scale administrative cohort data from Korea provide additional perspective. In a national birth cohort, children with CH had a higher risk of delayed development on the Korean ASQ (adjusted RR 2.77, 95% CI: 1.507–5.118 for total developmental area), with significant differences in gross motor, fine motor and problem‐solving domains [20]. In a broader longitudinal analysis, CH children had a higher risk of suspected neurodevelopmental disorders (PS‐weighted OR: 4.52, 95% CI: 2.91–7.02), with significantly increased risk across all five K‐ASQ domains and delayed medication was related to worse outcomes [21].

The availability of published literature employing ASQ‐3 as a neurodevelopmental screening tool in CH cohorts supported its use in our study as a feasible and validated alternative to formal psychometric testing, which was not possible due to cost constraints. A meta‐analysis of IQ outcomes in early‐treated CH found that IQ scores were within normal limits but significantly lower than controls, with pooled mean differences of −9.05 for verbal IQ, −11.70 for performance IQ and −10.78 for total IQ [25]. In a Brazilian 25‐year cohort (*n* = 458), FSIQ scores correlated with T4 at diagnosis and maternal education, with 11.6% of participants having intellectual deficit, providing evidence that hypothyroidism severity is a strong determinant of childhood cognitive level [26].

In a Dutch cohort, patients with severe CH had lower full‐scale (93.7), verbal (94.9) and performance (93.9) IQ scores, with significant motor problems across all severity subgroups [27]. The social context can confound these relationships; a Chilean study found no cognitive differences between CH children and controls after accounting for socioeconomic status, with higher family income and more assets predicting better performance regardless of CH status [28]. Outcomes are substantially worse, where NBS is absent or diagnosis is established late. In Algeria, where median age at diagnosis was 1.6 months and only 28% started treatment before 30 days, mean IQ was 86.1% and 11% had IQ < 70 [29]. In Indonesia, where NBS had not been established, 18 of 25 clinically diagnosed patients had a total IQ < 70, and late initiation of thyroid hormone substitution negatively affected intellectual abilities [30]. In Pakistan's non‐screened clinical setting, a Multan‐based study of 119 CH children found high rates of developmental delay across all Denver Developmental Screening Test (DDST) domains, and children who started thyroid replacement within 2 months had lower frequency of significant delay than those who started later. Yet, only 46.2% of children had started treatment within 2 months of life [31]. Although our patients had normal ASQ scores in 34 children, formal IQ testing may help to identify IQ delays more accurately.

Across the literature, earlier treatment initiation is repeatedly linked to improved outcomes. Selva et al. showed that subjects started on higher initial levothyroxine doses (50 μg) had full‐scale IQ scores 11 points higher than those on lower initial doses, and taking longer than 2 weeks to normalize thyroid function was associated with significantly lower cognitive, attention and achievement scores [9]. In a Portuguese cohort, later treatment initiation was negatively correlated with performance IQ (*r* = −0.50, *p* = 0.028) and perceptual organization index (*r* = −0.57, *p* = 0.022), and elevated rates of ADHD (26.1%) and learning disorders (19.6%) were documented even in treated children [8]. These findings reinforce that early treatment initiation and adequacy of initial dosing remain clinically actionable determinants of outcome.

This study has several strengths. It is the first report from Pakistan describing growth and neurodevelopmental outcomes of CH children identified through a structured DBS‐based NBS programme. The use of ASQ‐3, an internationally validated, widely used developmental screening tool, allows direct comparison with published literature. The five‐year study period provides a reasonable cohort for a single‐centre programme. However, several limitations must be acknowledged. The study is retrospective and from a single tertiary urban centre, which may limit generalizability to rural Pakistan or other settings. There were no matched healthy controls, precluding direct comparison. Ages at ASQ‐3 assessment varied, and only the last recorded assessment was used, rather than serial longitudinal assessments. The ASQ‐3 is not a formal psychometric test such as the Bayley Scales or Wechsler Preschool and Primary Scale of Intelligence; however, the availability of published literature employing ASQ‐3 as a neurodevelopmental screening tool in CH cohorts supported its use in our study as a feasible and validated alternative to formal psychometric testing, which was not possible due to cost constraints. Important confounders including socioeconomic status, parental education, treatment adherence and levothyroxine dose adequacy were not systematically analysed due to very small sample size. Loss to follow‐up may introduce survivor bias.

## 5 Conclusions

The incidence of CH is estimated to be considerably higher in Pakistan than global averages. This study demonstrates that a structured DBS‐based NBS program can achieve high screening coverage and enable early identification and treatment of affected children. Our growth findings suggest that early‐treated CH children can achieve near‐normal growth parameters. Our ASQ‐3 findings indicate that the majority of early‐treated children demonstrate normal development. These findings are consistent with international evidence showing that early detection and adequate levothyroxine treatment substantially improve neurodevelopmental outcomes, while also highlighting that developmental surveillance remains important even in treated cohorts. Pakistan urgently needs a national NBS program, strengthened screening‐to‐treatment cascades, and structured developmental follow‐up pathways to prevent the avoidable neurodevelopmental consequences of delayed CH diagnosis.

## Author Contributions

**Bushra Rehman:** conceptualization, methodology, formal analysis, supervision, writing – review and editing, writing – original draft, project administration, visualization, validation. **Muhammad Nasheet Sagri:** conceptualization, writing – review and editing. **Fozia Memon:** investigation, data curation, formal analysis. **Farzana Faqiruddin:** writing – original draft. **Salman Kirmani:** validation. **Hafsa Majid:** supervision. **Sumaiyah Zahid:** methodology, formal analysis. **Nadia Mazhar:** writing – review and editing. **Muzna Arif:** validation, visualization, formal analysis. **Zahra Hoodbhoy:** software, resources. **Khadija Nuzhat Humayun:** formal analysis. **Abida Akbar:** investigation, writing – original draft, project administration.

## Funding

The authors have nothing to report.

## Ethics Statement

The study was conducted in accordance with the Declaration of Helsinki, and full review was waived due to the retrospective study design and the use of anonymized medical records. (ERC approval number: 2024–10,452‐30,741, dated 19th September, 2024 by the Ethical Review Committee of Aga Khan University Hospital).

## Consent

The authors have nothing to report.

## Conflicts of Interest

The authors declare no conflicts of interest.

## Data Availability

The data that support the findings of this study are available on request from the corresponding author. The data are not publicly available due to privacy or ethical restrictions.
